# Integrating PICO principles into generative artificial intelligence prompt engineering to enhance information retrieval for medical librarians

**DOI:** 10.5195/jmla.2025.2022

**Published:** 2025-04-18

**Authors:** Kyle Robinson, Karen Bontekoe, Joanne Muellenbach

**Affiliations:** 1 krobinson@chsu.edu, Librarian Manager, Systems and Technical Services, Health Sciences Library, California Health Sciences University, CA; 2 kbontekoe@chsu.edu, User Services Librarian, Health Sciences Library, California Health Sciences University, CA; 3 jmuellenbach@chsu.edu, Director, Health Sciences Library, California Health Sciences University, CA

**Keywords:** PICO, Generative Artificial Intelligence, Prompt Engineering, Information Retrieval

## Abstract

Prompt engineering, an emergent discipline at the intersection of Generative Artificial Intelligence (GAI), library science, and user experience design, presents an opportunity to enhance the quality and precision of information retrieval. An innovative approach applies the widely understood PICO framework, traditionally used in evidence-based medicine, to the art of prompt engineering. This approach is illustrated using the “Task, Context, Example, Persona, Format, Tone” (TCEPFT) prompt framework as an example. TCEPFT lends itself to a systematic methodology by incorporating elements of task specificity, contextual relevance, pertinent examples, personalization, formatting, and tonal appropriateness in a prompt design tailored to the desired outcome. Frameworks like TCEPFT offer substantial opportunities for librarians and information professionals to streamline prompt engineering and refine iterative processes. This practice can help information professionals produce consistent and high-quality outputs. Library professionals must embrace a renewed curiosity and develop expertise in prompt engineering to stay ahead in the digital information landscape and maintain their position at the forefront of the sector.

## CURRENT CHALLENGES

The digital information landscape is undergoing rapid transformation, posing both opportunities and challenges for medical librarians. With the exponential growth of digital content and the increasing complexity of information needs, librarians are constantly seeking ways to enhance the precision and efficiency of information retrieval. The current challenges include:

1. **Information Overload:** The sheer volume of information available online can be overwhelming for users. Librarians must navigate this vast sea of data to find accurate, relevant, and timely information for their patrons [1].
2. **Evolving User Expectations:** Today's users demand instant access to information that is not only accurate but tailored to their specific needs. This shift in expectations requires librarians to adopt more sophisticated search techniques and tools [2].
3. **Integration of Advanced Technologies:** The integration of technologies such as artificial intelligence (AI), machine learning, and natural language processing into library systems presents both opportunities and hurdles. While these technologies can enhance information retrieval, they also require librarians to develop new skills and adapt to rapidly changing tools [3].
4. **Quality and Reliability:** Ensuring the quality and reliability of information remains a critical concern. Librarians must be adept at evaluating sources and filtering out misinformation, particularly in an era where digital content can be easily manipulated [4].

These challenges underscore the need for innovative approaches to information retrieval. By adopting and mastering new techniques such as prompt engineering, librarians can continue to meet the evolving needs of their patrons effectively.

## BACKGROUND

Generative Artificial Intelligence (GAI) is rapidly transforming the landscape of information retrieval. GAI refers to a class of artificial intelligence systems capable of generating new content, such as text, images, or music, by learning patterns from existing data. These systems use deep learning models to create outputs that can mimic human-made content [5]. With the advent of sophisticated GAI technologies, the ability to generate high-quality, contextually relevant responses has become increasingly critical. From the first known bibliographic work, the *Pinakes*, created in the Library of Alexandria in the 3rd century BCE [6], to the development of machine-readable cataloging (MARC) standards in the 1960s [7], and through modern challenges like open access, data management, and digital preservation, librarians have consistently been at the forefront of informational revolutions throughout history [8]. The current transformation driven by GAI, though in its infancy, is unprecedented in its rapid expansion and impact. Once again, librarians and information professionals are tasked with leveraging advancements created by a technological evolution to meet the evolving needs of their patrons.

Prompt engineering is an emerging practice within the GAI field that focuses on designing effective prompts to guide artificial intelligence (AI) systems in generating useful and accurate responses. At its core, prompt engineering involves the careful construction of inputs or instructions that an AI system uses to produce relevant and high-quality information outputs. The process is akin to asking a detailed question or setting a clear task for the AI, which, in turn, generates an answer or a piece of content that meets the specified requirements [9].

The practice of prompt engineering can be compared to formulating precise search queries or research questions, but it goes beyond keyword matching. Instead, it involves a nuanced approach that considers the context, desired output, and the user's specific needs. This makes it a powerful tool for guiding AI systems to deliver more accurate, contextually appropriate, and relevant information [10].

Effective prompt engineering requires a deep understanding of both the subject matter and the capabilities of the AI system. It encompasses various elements, such as the needed task, the context in which the information will be used, and the audience for which the output is intended. For instance, a well-crafted prompt for a GAI system might specify not only the topic of interest but also the type of information required, an example to help guide the output, the format in which it should be presented, and the tone that is appropriate for the intended audience.

The significance of prompt engineering lies in its ability to enhance the precision and quality of information retrieval. In an era of information overload, where the volume of available data can be overwhelming, effective prompt engineering enables users to obtain more focused and relevant outputs from AI systems. This not only improves the efficiency of information retrieval but also ensures that the information generated is aligned with the user's specific needs and context.

## TECHNOLOGICAL ADVANCES

The evolving role of prompt engineering is a critical component of modern information retrieval. It facilitates the creation of accurate and contextually relevant prompts that address specific user needs, thereby improving the quality and precision of AI-generated responses. This parallels traditional practices such as the use of controlled vocabularies and tailored search strategies, emphasizing the necessity for librarians to adapt these skills to the capabilities of GAI technologies [11]. By mastering prompt engineering, librarians can effectively navigate the complexities of information overload and enhance the retrieval processes of GAI platforms.

The current era is marked by innovative technological advances that are reshaping the information retrieval field. Among these, GAI stands out as a transformative force. GAI technologies leverage deep learning algorithms to generate human-like text and have the potential to revolutionize how librarians interact with and retrieve information [12]. However, GAI is just one part of a broader technological landscape that is influencing library and information science. Other notable advancements, many of which paved the way for GAI, include:

1. **Natural Language Processing (NLP):** NLP technologies enable computers to understand and process human language, improving the accuracy and relevance of search results. These tools allow for more intuitive interactions with information systems, making it easier for users to find what they need [13].
2. **Machine Learning:** Machine learning algorithms analyze patterns in data to improve search results over time. By learning from user interactions, these systems can provide increasingly personalized and relevant information [13].
3. **Semantic Search:** Unlike the traditional keyword-based search, semantic search aims to understand the intent behind a query. This approach leads to more accurate and contextually appropriate results, enhancing the user experience [14].
4. **Voice-Activated Search:** The rise of voice-activated assistants like Siri, Alexa, and Google Assistant has provided new ways for users to interact with information systems. This technology relies on sophisticated AI and NLP to understand and respond to voice queries accurately [15].
5. **Blockchain for Information Integrity:** Blockchain technology offers a promising solution for ensuring the integrity and authenticity of digital information. By creating immutable records, blockchain can help combat misinformation and provide verifiable sources [16].

These technological advances are, arguably, driving a paradigm shift in information retrieval. As librarians embrace these new tools, they must also develop new competencies and evolve foundational skill sets to leverage their full potential [11]. The integration of traditional frameworks such as PICO into prompt engineering represents one such pathway, enabling librarians to harness the power of GAI and other technologies effectively but in a way that builds upon current experience. By staying at the forefront of these developments and using familiar tools, librarians can continue to play a critical role in guiding users through the complexities of the digital information landscape.

## APPLICATION OF PICO PRINCIPLES

The PICO framework (Population, Intervention, Comparison, Outcome) has been a cornerstone in medical research for structuring research questions and guiding systematic reviews. By breaking down complex questions into clear, manageable components, PICO can facilitate targeted and effective information retrieval. This systematic approach has proven invaluable in medical research, providing a structured methodology for acquiring and synthesizing information [17].

Integrating these standardized principles into prompt engineering may enhance the precision and relevance of responses while also providing a standardized approach that can be adapted across various prompt frameworks depending on the desired outcome. This standardization is essential for ensuring consistency and quality in information retrieval, enabling librarians and information professionals to deliver high-quality outputs consistently and efficiently. The application of the PICO framework's systematic approach to prompt engineering represents an important advancement in information science. By leveraging frameworks like TCEPFT, librarians and information professionals can develop more effective and precise prompts, thereby improving the overall quality of information retrieval and enhancing their role as key players in the information landscape.

### Possible Benefits of PICO-Driven Prompt Engineering

1. Enhanced Precision: Applying PICO principles helps define clear, specific tasks that are contextually relevant and personalized.
2. Consistency: Standardized frameworks ensure that prompts produce high quality results across different use cases and contexts.
3. Flexibility: The modular nature of PICO allows for easy adjustments and refinements to prompts, ensuring they remain relevant to changing needs.

### Practical Application

Consider this scenario:

A physician needs information about complications specific to type 2 diabetes in elderly patients.

Combining the TCEPFT framework with PICO principles, the prompt could be structured as follows:

**Table:** 

**Task**	Identify common complications.
**Context**	Specific to type 2 diabetes in elderly patients.
**Example**	Such as complications that impact mobility or daily activities.
**Persona**	For a primary care physician preparing for patient consultations.
**Format**	Detailed paragraph with clear subheadings for each complication.
**Tone**	Professional and thorough.

Once the framework is established and the components defined, the prompt is easily constructed similarly to how a research question is formulated from a PICO instance. The arrangement should follow a logical flow, resulting in a prompt such as:

**Prompt:** “Identify the most common complications of type 2 diabetes in elderly patients that affect mobility or daily activities, presented in a detailed paragraph with subheadings for each complication. This information is for a primary care physician to prepare for patient consultations, so please maintain a professional and thorough tone.”

This structured approach ensures that the generated output is precise, relevant, and tailored to the physician's needs.

To reinforce this idea, consider another scenario:

A medical librarian gets a request from a medical school faculty member preparing a lecture on the impact of sleep disorders on mental health in adolescents. The faculty member needs comprehensive and up-to-date information to ensure the lecture is evidence-based and informative.

Using the TCEPFT framework, the librarian can structure the prompt as follows:

**Table:** 

**Task**	Gather information on the impact of sleep disorders on mental health in adolescents.
**Context**	Specific to sleep disorders such as insomnia and sleep apnea and their psychological effects like anxiety and depression.
**Example**	Focus on studies that explore the correlation between sleep disorders and mental health outcomes in adolescent populations.
**Persona**	For a medical faculty member preparing an evidence-based lecture for medical students.
**Format**	A detailed summary with key findings from recent research studies, organized by type of sleep disorder.
**Tone**	Academic and informative.

**Prompt:** “Identify and summarize the latest research on the impact of sleep disorders, such as insomnia and sleep apnea, on mental health outcomes in adolescents, specifically focusing on anxiety and depression. Present the findings in a detailed summary, organized by type of sleep disorder. This information is intended for a medical faculty member preparing an evidence-based lecture for medical students, so the tone should be academic and informative.”

### Flexibility in Prompt Engineering

Another benefit of using a structured approach is the ability to switch out components without the need to recreate a prompt entirely. While the output will never be the same, it will be similar in structure and presentation. This flexibility allows medical librarians to adapt prompts to meet varying information needs efficiently.

For example, consider the first scenario where the prompt is focused on complications specific to type 2 diabetes. By changing “type 2 diabetes” to “chronic kidney disease,” the content of the output will certainly differ, but the overall structure, audience, and tone will remain consistent. This ensures that the librarian can quickly modify the prompt to address a new yet related information request without starting from scratch.

In another scenario, switching out “sleep disorder” for “drug abuse” would again alter the content but maintain the same structure and presentation style. This adaptability is imperative for medical librarians who frequently receive requests with similar needs but slightly different variables. For instance, a request for information on the impact of sleep disorders on mental health in adolescents can be easily adjusted to focus on the impact of drug abuse on mental health by simply changing the specific condition in the prompt.

Here is an example of how this flexibility works:

- **Original Prompt for Sleep Disorders:** “Identify and summarize the latest research on the impact of sleep disorders, such as insomnia and sleep apnea, on mental health outcomes in adolescents, specifically focusing on anxiety and depression. Present the findings in a detailed summary, organized by type of sleep disorder. This information is intended for a medical faculty member preparing an evidence-based lecture for medical students, so the tone should be academic and informative.”
- **Modified Prompt for Drug Abuse:** “Identify and summarize the latest research on the impact of drug abuse, such as the use of opioids and cannabis, on mental health outcomes in adolescents, specifically focusing on anxiety and depression. Present the findings in a detailed summary, organized by type of substance. This information is intended for a medical faculty member preparing an evidence-based lecture for medical students, so the tone should be academic and informative.”

By altering the specific condition (sleep disorders to drug abuse), the librarian can generate a relevant and tailored output without the need to reconstruct the entire prompt.

## CONCLUSION

The integration of the PICO framework into prompt engineering methodologies presents an important advancement for medical librarians and information professionals. By adopting a structured approach like the TCEPFT framework, librarians can enhance the precision, consistency, and flexibility of information retrieval processes. This systematic method not only improves the quality of the outputs but also ensures that librarians can efficiently adapt to various information needs, thereby meeting the evolving demands of their patrons. Consistency is more easily attainable if the process used is already a familiar staple in day-to-day work, as is the case with PICO and medical librarians.

It is important to acknowledge that the TCEPFT framework is not the only way to approach prompt engineering. There are numerous other variations and frameworks that can be equally effective, depending on the context and specific requirements of the task at hand. Additionally, not every situation may necessitate a full structure like TCEPFT. In some cases, a simpler or more intuitive approach might be more appropriate. However, maintaining consistency in the application of whichever framework is chosen is essential for producing high-quality and reliable results. In essence, while the framework of the initial prompt may be fluid based on the desired outcome, the process and step logic to get to a quality prompt can be invoked by calling upon an already established and typically honed skill that medial librarians possess.

As the field of librarianship continues to intersect with advanced technologies like GAI, it is imperative for librarians to explore and master these new tools. By doing so, they can remain at the forefront of the digital information landscape, providing valuable services and support to their patrons. As has been the case throughout history, library professionals have a unique opportunity to cultivate a renewed curiosity and actively engage with burgeoning information technology, which currently is focused on GAI technologies and prompt engineering techniques. Embracing these innovations will enhance their skill sets and ensure that they continue to play a pivotal role in the future of information retrieval.
